# Integrated proteomic and metabolomic analysis reveals the mechanism of astragalus complanatus flavonoids against liver fibrosis in rats

**DOI:** 10.3389/fphar.2026.1949321

**Published:** 2026-09-28

**Authors:** Ruina Zhou, Yang Wang, Xinbo Shi, Shujie Peng, Xiaoni Kou, Gaoying Yan, Lin Chen, Min Cheng

**Affiliations:** 1 Co-construction Collaborative Innovation Center for Chinese Medicine Resources Industrialization by Shaanxi and Education Ministry, Shaanxi University of Chinese Medicine, Xianyang, China; 2 Department of Pharmacology, Shaanxi University of Chinese Medicine, Xianyang, China; 3 Affiliated Hospital of Shaanxi University of Traditional Chinese Medicine, Xianyang, China; 4 Shaanxi Lijun Modern Traditional Chinese Medicine Co., Ltd., Xi’an, China; 5 College of Modern Agriculture and Bioengineering, Shangluo University, Shangluo, China

**Keywords:** Astragalus complanatus flavonoids, integrated analysis, liver fibrosis, metabolomics, proteomics

## Abstract

**Objective:**

Astragali Complanati Semen (ACS) is a traditional Chinese medicinal herb conventionally applied to tonify the liver and kidney. As the primary bioactive ingredients of ACS, Astragalus complanatus flavonoids (ACF) exert hepatoprotective and anti-fibrotic effects. This study aimed to elucidate the antifibrotic mechanisms of ACF through integrated proteomic and metabolomic analyses combined with experimental validation.

**Methods:**

A CCl_4_-induced rat model of LF and TGF-β1-activated hepatic stellate cell (HSC) model were established to evaluate the antifibrotic effects of ACF. Integrated proteomic and metabolomic analyses were performed to identify differentially expressed proteins (DEPs), differentially expressed metabolites (DMs), and associated signaling pathways. Key molecular targets were validated using quantitative real-time PCR and Western blotting, while pharmacological inhibitors were employed to investigate the involvement of the FAK/AKT signaling pathway in ACF-mediated inhibition of HSC activation.

**Results:**

Compared with the LF model group, ACF treatment significantly ameliorated liver function indicators, relieved histopathological injury and collagen deposition, decreased serum fibrosis markers, downregulated the protein levels of Col I, Col IV and α-SMA, and reduced oxidative stress and inflammatory response. Proteomic screening uncovered 401 differentially expressed proteins (DEPs), which were primarily enriched in extracellular matrix (ECM)–receptor interaction, focal adhesion and PI3K/AKT signaling pathways. Protein–protein interaction analysis further pinpointed FN1, CD44, ITGAV, FAK and AKT1 as core target proteins. In vitro experiments demonstrated that ACF blocked TGF-β1-triggered HSC activation, lowered the expression of α-SMA and ITGAV, and inhibited the phosphorylation of FAK and AKT. Pharmacological blockade of either FAK with PF-562271 or AKT with MK2206 strengthened the anti fibrogenic activity of ACF, verifying that the FAK/ AKT axis mediates the anti-HSC activation effect of ACF. Metabolomic analysis identified 108 differentially expressed metabolites (DMs), mainly enriched in glutathione metabolism, primary bile acid biosynthesis and carbohydrate metabolism. Integrated multi-omics analysis confirmed close correlations between DEPs and DMs within ECM–receptor interaction, PI3K/AKT signaling and primary bile acid biosynthesis pathways. Specifically, glycochenodeoxycholic acid, dulcitol and chenodeoxycholate showed strong correlations with coretarget proteins.

**Conclusion:**

ACF achieves comprehensive anti-LF effects via suppressing FAK/AKT1 signaling to restrain HSC activation, while coordinately modulating pivotal metabolic pathways.

## 1 Introduction

According to age-standardized mortality data published by the Global Burden of Disease Study collaborators (2024), cirrhosis became the 12th leading cause of death worldwide in 2021, posing a significant threat to human health (Collaborators, 2024). Liver fibrosis (LF) is a key pathological step in the evolution of chronic hepatic disease toward cirrhosis; various risk factors, including exposure to toxins, chronic heavy drinking, viral hepatitis infection, and a high-fat diet, can all contribute to the development of LF. From a pathological perspective, the essence of LF lies in the excessive production and abnormal deposition of the extracellular matrix (ECM), which triggers hepatic fibrous scar formation and slowly destroys the regular microarchitecture (Kisseleva and Brenner, 2021; Yao et al., 2024). The early clinical symptoms of LF primarily include fatigue, weakness, loss of appetite, changes in bowel habits, and vague pain or discomfort in the right upper abdomen. These nonspecific symptoms make early diagnosis challenging and increase the risk of the disease progressing unnoticed.

Extensive research has demonstrated that LF is reversible in its early and even intermediate stages. However, without timely and effective intervention, the ongoing fibrotic process will progressively worsen the disruption of liver microstructure, leading to a progressive decline in liver function and ultimately progressing to cirrhosis, hepatocellular carcinoma, and even multi-organ failure—all of which are end-stage conditions closely associated with extremely high mortality rates (Hernandez-Gea and Friedman, 2011; Khurana et al., 2021). Therefore, implementing efficient clinical interventions during the LF stage is of significant clinical importance. However, to date, no therapeutic agent has been officially licensed worldwide for the specific management of LF. While some promising candidate compounds, such as belaprost, ceniverol, elafibron and imicasan, have entered Phase II/III clinical trials, their efficacy and safety have not yet been fully validated (Shan et al., 2022). The core reason for this challenge lies in the highly complex and multifactorial nature of the pathogenesis of LF—involving inflammatory responses, oxidative stress, metabolic disorders, extracellular matrix remodeling, and the interactive regulation of multiple cellular signaling pathways. Intervention strategies targeting a single pathway often fail to achieve optimal therapeutic outcomes. Consequently, most anti-fibrotic drugs that have entered clinical development have ultimately failed to gain validation, further highlighting the unmet need to deeply dissect the molecular mechanisms of LF and, based on this understanding, develop safe, effective, and multi-target synergistic intervention strategies. Future research should place greater emphasis on integrating multi-omics data at the systems biology level to explore the unique advantages of complex systems, such as traditional Chinese medicine, in regulating the fibrosis network, thereby providing new breakthroughs for the clinical treatment of LF (Lu et al., 2025).

Traditional Chinese Medicine (TCM), with its characteristic synergistic effects based on “multiple components, multiple targets, and multiple pathways,” as well as its core advantages of holistically regulating the body’s condition and reducing the side effects associated with single-target interventions, has been commonly used in hepatic disorders (Wei et al., 2022; Xu et al., 2024; Zhou et al., 2024). *Astragalus complanatus Semen* (ACS), the dried mature seeds of *Astragalus complanatus* R. Br., is predominantly cultivated in Shaanxi, Henan, and Hebei provinces of China. It was first documented in Compendium of Materia Medica (Ben Cao Gang Mu), the iconic Ming Dynasty pharmacopoeia compiled by Li Shizhen, and has been traditionally used to tonify the liver and kidney as well as improve visual acuity (Ng et al., 2014). The main constituents of ACS are flavonoids, amino acids, triterpenes, and organic acids, among which Astragalus complanatus flavonoids (ACF) are the major active components with extensive pharmacological properties, encompassing hepatoprotective and antifibrotic activities (Xue et al., 2008; Ng et al., 2014; Sham et al., 2017). Although ACF has an anti-LF effect in rats, the molecular mechanisms—especially the specific signaling pathways and key molecular targets involved—still require further elucidation (Liu et al., 2005). Proteomics and metabolomics approaches are the powerful analytical tools for deciphering the complex mechanisms of action inherent to TCM. In a rat model of LF, 4D label-free quantitative proteomics and untargeted metabolomics, combined with multi-omics integrative analysis, were employed to systematically identify the key differentially expressed proteins (DEPs) and differential metabolites (DMs) regulated by ACF. Key signaling pathways and molecular targets were verified *in vivo* and *in vitro*. This study systematically elucidated the mechanisms of ACF against LF at both the protein and metabolic levels, providing experimental evidence to support its clinical translation.

Previous studies have shown that ACF exerts a protective effect against chronic liver injury and liver fibrosis through its antioxidant, anti-inflammatory, and extracellular matrix-modulating activities. It has been reported that, in experimental models, ACF treatment can improve liver dysfunction, alleviate oxidative stress, and inhibit the fibrotic response (Liu et al., 2005). However, most previous studies have focused only on a single biological marker or a single signaling pathway, which may not fully explain the complex pharmacological properties of ACF. Flavonoids are multifaceted and multitarget compounds; the comprehensive molecular mechanisms underlying ACF’s antifibrotic effects remain unclear, and the interactions between protein-level regulatory changes and metabolic remodeling during ACF intervention have not yet been systematically elucidated. Therefore, identifying the key molecular targets, metabolic pathways, and integrated regulatory networks involved in ACF-mediated antifibrotic effects represents an important scientific question.

## 2 Materials and methods

### 2.1 Drugs and materials

ACS (batch No. S20210102) were acquired from Shaanxi Bainshou Pharmaceutical Co., Ltd. (Shaanxi, China). ACF were extracted in the laboratory. Briefly, 2 kg of ACS were pulverized and extracted twice with 50% methanol. The pooled filtrates were concentrated to yield 430 g of crude extract, which was then solubilized in warm water, filtered, and applied to 2 kg of HPD-100A macroporous adsorption resin. The column was washed sequentially with water and 70% methanol. The latter fraction was collected, concentrated, and dried, yielding 43.68 g of ACF. Silymarin and carbon tetrachloride (CCl_4_) were supported by Taisili Pharmaceutical Co., Ltd. (Tianjin, China).

### 2.2 Identification and analysis of ACF extract components

ACF powder was dissolved in 50% methanol to obtain a 4 mg/mL working solution. UPLC separation used an Agilent ZORBAX SB-C18 column (2.1 × 100 mm, 1.8 μm) with mobile phases A (acetonitrile) and B (0.1% formic acid) under gradient elution (Supplementary Table S1). Flow: 0.3 mL/min; injection: 3 μL. MS detection used a Waters G2 QTOF with ESI (±) mode (m/z 100–1500). The total flavonoid content of ACF was additionally determined by UV–visible spectrophotometry using rutin as the reference standard.

### 2.3 Animal handling and drug treatment

Male Sprague Dawley (SD) rats (6–8 weeks, 180 ± 20 g) were supported by Chengdu Dashuo Experimental Animal Co., Ltd. (SCXK (Chuan) 2020–030). All procedures complied with the Animal Experiment Guidelines of Shaanxi University of Chinese Medicine and were approved by the Institutional Ethics Committee (No. SUCMDL20221017001).

LF was induced in model, silymarin, ACF 40, and ACF 80 groups by i. p. Injection of 40% CCl_4_ (2 mL/kg) twice weekly for 8 weeks (Figure 1). Controls received equal volume of olive oil i. p. From week 3 onward, silymarin (100 mg/kg) or ACF (40/80 mg/kg) was given daily by gavage; model and control groups received normal saline orally. After 8 weeks, following a 12-h fast (water *ad libitum*), blood and liver tissues were collected.

**FIGURE 1:**
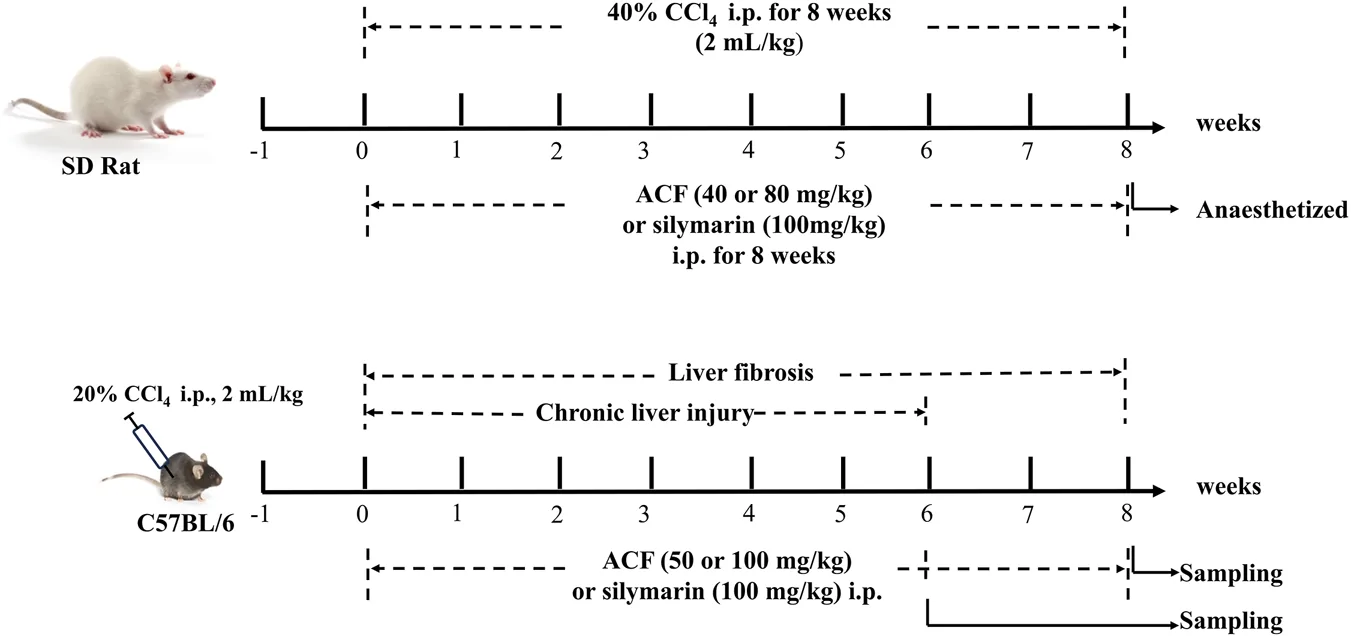
Schematic of the animal experiment.

### 2.4 Histopathological examination and parameters analysis

Liver specimens were processed for hematoxylin and eosin (HE) and Masson’s trichrome staining to assess histopathological alterations. Serum AST, ALT, ALP, ALB, and TBA were determined by a biochemical analyzer. SOD activity and GSH and HYP levels were measured using commercial kits. IL-6, IL-1β, TNF-α, Col-IV, HA, LN, and PCIII were measured by ELISA method.

### 2.5 Immunohistochemical staining

FN1 and α-SMA were semi-quantitatively analyzed by immunohistochemical staining. For each stained section, the positive staining area was measured in multiple random fields using ImageJ software. The proportion of positively stained regions was determined using the equation outlined below:

$$\text{Positive area }\left(\%\right)=\left(\text{Positive staining area }/\text{ Total tissue area in the field}\right)\times 100\%$$

### 2.6 Proteomics analysis

Protein identification and quantification were conducted by MaxQuant software (version 1.6.14). Absolute protein quantification was achieved by normalizing total peak intensities and matched peptide counts against the Ensembl_Rattus database. Differential expression analysis was performed based on quantitative protein data; DEPs were identified according to fold change (FC > 1.5 or <0.66), *P* < 0.05, and Benjamini–Hochberg false discovery rate (FDR)-adjusted *P* < 0.05. Multiple testing correction was performed using the Benjamini–Hochberg procedure.

### 2.7 qRT-PCR analysis

Total RNA was extracted using the TRIzol method, with at least three independent replicates. GAPDH was used as the internal reference gene. The Ct values of AKT1, FAK, FN1, ITGAV, and CD44 were normalized to GAPDH, and relative mRNA expression was calculated using the 2^−ΔΔCt^ method, and quantified using the Q Tower 2.2 system (Analytik Jena). Detailed primer sequences are shown in Table 1.

**TABLE 1:** Primer sequences.

Gene	Forward primer (5′-3′)	Reverse primer (5′-3′)
AKT1	ATG​AGC​GAC​GTG​GCT​ATT​GTG​AAG	GAG​GCC​GTC​AGC​CAC​AGT​CTG​GAT​G
FAK	TGAGCCACTGGCCATTGA	GCT​GCT​GGT​GGA​ATG​CTA​GAG
FN1	CTG​GTT​ACC​CTT​CCA​CAC​CC	GGT​GAC​GAA​GGG​GGT​CTT​TT
ITGAV	ATA​AAG​CGC​GGA​TGG​CAA​AG	CTC​ACC​CGA​AGA​TAG​GCG​AC
CD44	TCG​ATT​TGA​ATG​TAA​CCT​GCC​G	CAG​TCC​GGG​AGA​TAC​TGT​AGC
GAPDH	TGC​ACC​ACC​AAC​TGC​TTA​G	GGA​TGC​AGG​GAT​GAT​GTT​C

### 2.8 Western blotting

Total protein was purified from liver tissue. After SDS-PAGE and PVDF blotting, membranes were blocked and probed overnight at 4 °C with primary antibodies (GAPDH 1:50,000; AKT1 1:1,000; p-AKT1 1:750; FAK 1:5,000; collagen I/IV 1:2,000 & 1:1,000; TGF-β1 1:2,000; CD44 1:500). Immunoreactive signals were detected via ECL and quantified with Image Lab.

### 2.9 Cell culture and drug treatment

HSC-T6 was purchased from Beijing Beichuanglian Biotechnology Research Institute (Beijing, China). Cells were maintained in high-glucose DMEM with 10% FBS and 1% penicillin-streptomycin. When cell confluence reached approximately 70%, the cells were treated as follows: stimulation with TGF-β1 (10 ng/mL) alone, or co-treatment with TGF-β1 combined with ACF (at specific concentrations), the FAK inhibitor PF-562271 (1 μM), or the AKT inhibitor MK2206 (1 μM). Cultivation was continued for 24 h before subsequent analysis.

### 2.10 Cell viability assay

HSC-T6 cells in the logarithmic phase of proliferation were dispensed into 96-well microplates at a density of 5 × 10^4^ cells/mL. Once the cultures attained 70% confluency, they were subjected to graded concentrations of ACF for a 24-h incubation period. Cellular viability was subsequently quantified using a CCK-8 kit, adhering strictly to the manufacturer’s specifications. In a parallel arm of the experiment, HSC-T6 cells (at 70% confluence) were initially pre-exposed to TGF-β1 (10 ng/mL) for 24 h to instigate activation, followed by a further 24-h challenge with varying doses of ACF. The proportion of viable cells was then assessed via the CCK-8 procedure.

### 2.11 Western blotting analysis of HSC-T6 cells

Total protein was purified from HSC-T6 cells and measured by BCA assay. Primary antibodies used for Western blotting included: anti-GAPDH (1:50,000), anti-AKT1 (1:1,000), anti-phosphorylated AKT1 (1:750), anti-FAK (1:5,000), anti-phosphorylated FAK (1:5,000), anti-α-SMA (1:50,000), and anti-integrin αV (ITGAV; 1:1,000). Immunoreactive signals were detected via ECL and quantified with Image Lab.

### 2.12 Metabolomics analysis

Raw UPLC-MS data were processed, and DMs were selected based on the following criteria: variable importance in projection (VIP) value >1, FC > 1.5 or <0.66, *P* < 0.05, and FDR-adjusted *P* < 0.05. The Benjamini–Hochberg method was applied for multiple testing correction. The selected DMs were uploaded to MetaboAnalyst, and functional and annotation information was analyzed using public databases (HMDB, LipidMaps, MassBank, mzCloud, and KEGG).

### 2.13 Multi-omics integrative analysis

To further elucidate relationships among different biological entities, pathway enrichment analysis was conducted using the MetaboAnalyst online tool, and multi-omics data were analyzed by joint pathway analysis. Under identical experimental conditions, the proteomics and metabolomics expression results were subjected to integrated metabolic pathway analysis. Advanced chord diagrams and correlation network plots were generated to visualize the correlations.

### 2.14 Statistical analysis

Statistical analyses were performed using IBM SPSS 26.0 and GraphPad Prism 8. Data are expressed as mean ± SD. All experiments were independently repeated at least three times. For comparisons among multiple groups, one-way ANOVA followed by Tukey’s *post hoc* test was used. qRT-PCR data were analyzed based on relative expression values calculated using the 2^−ΔΔCt^ method. For proteomics and metabolomics datasets, differential molecules were identified based on fold change, statistical significance, and Benjamini–Hochberg false discovery rate correction. FDR-adjusted *P* values <0.05 were considered statistically significant.

## 3 Results

### 3.1 Characterization of chemical components in ACF using UPLC–Q-TOF/MS

Data was carried out with Analyst TF 1.7.1 software and PeakView 1.2 software. Retention time served as the basis for initial compound assignment, followed by further verification through precursor and fragment ion analysis together with published literature. As shown in Figure 2 and Table 2, a set of 22 constituents was successfully characterized in the ACF sample, including 2-phenylethyl β-primeveroside, myricetin-3-rutinoside, myricetin 3-O-β-D-glucopyranoside, quercetin 3-O-sambubioside, camelliaside B, hyperin, cannabiscitrin, laricitrin 3-O-glucoside, complanatoside B, complanatuside, tectorigenin 7-O-xylosylglucoside, and comploside II, etc. (Figure 2; Table 2). Furthermore, the total flavonoid content of ACF was determined by UV–visible spectrophotometry using rutin as the reference standard. The total flavonoid content of ACF was 77.25%, expressed as rutin equivalents.

**FIGURE 2:**
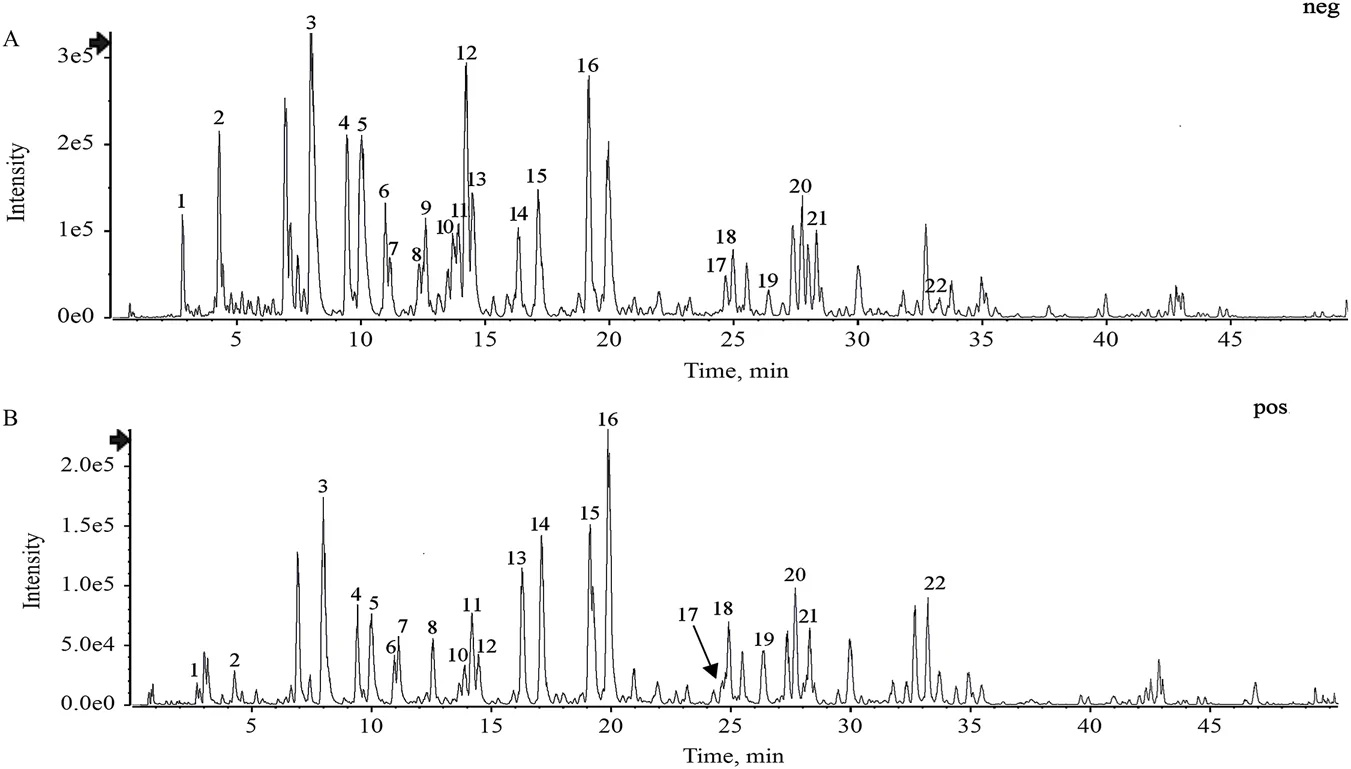
UPLC–Q-TOF/MS base peak ion chromatogram of ACF. **(A)** Negative ion spectrum. **(B)** Positive ion spectrum.

**TABLE 2:** Components of ACF determined on the basis of characterization and a database search.

NO	Retention time (min)	Adduct ion	m/z actual value	m/z theoretical value	Molecular formula	Molecular weight	Compound name	MS/MS peaks	Reference
1	2.82	[M + FA-H]^-^	447.1543	447.1508	C_18_H_26_O_10_	402.15	Benzyl β-primeveroside	401.1461; 269.1035; 161.0459; 101.0244	(Su et al., 2021)
2	4.31	[M + FA-H]^-^	461.1682	461.1665	C_19_H_28_O_10_	416.17	2-Phenylethyl β-primeveroside	415.1632; 311.0997; 191.0572; 149.0465; 131.0360; 89.0255	(Su et al., 2021)
3	8.02	[M-H]^-^	611.1284	611.1254	C_26_H_28_O_17_	612.13	Myricetin 3-O-β-D-xylopyranosyl (1–2)-β-D-glucopyranoside	611.1258; 316.0217; 287.0194; 271.0250; 178.9986	(Xiaozhou Jia et al., 2024)
4	9.46	[M-H]^-^	625.1464	625.1410	C_27_H_30_O_17_	626.15	Myricetin-3-rutinoside	625.1424; 316.0224; 287.0204; 271.0255; 178.9988	(Kuo et al., 2020)
5	10.04	[M-H]^-^	479.0872	479.0872	C_21_H_20_O_13_	480.09	Myricetin 3-O-β-D-glucopyranoside	479.0879; 316.0230; 287.0189; 271.0243; 178.9985	(Qi et al., 2011)
6	10.99	[M-H]^-^	595.1340	595.1305	C_26_H_28_O_16_	596.14	Quercetin 3-O-sambubioside	595.1330; 300.0274; 271.0235; 255.0287; 178.9977	(Guo et al., 2023)
7	11.16	[M + FA-H]^-^	491.1229	491.1229	C_22_H_22_O_10_	446.12	Calycosin-7-O-β-D-glucoside	491.1206; 283.0605; 268.0372	(Guan et al., 2022)
8	12.35	[M-H]^-^	449.0737	449.0725	C_20_H_18_O_12_	450.08	Myricetin-3-O-β-D-Xylopyranoside	449.0747; 316.0217; 287.0195; 271.0240; 178.9981	(Vvedenskaya et al., 2004)
9	12.61	[M-H]^-^	725.1976	725.1935	C_32_H_38_O_19_	726.20	Camelliaside B	725.1959; 575.1442; 284.0329; 255.0296; 227.0357	(Liu et al., 2018)
10	13.71	[M-H]^-^	463.0889	463.0882	C_21_H_20_O_12_	464.10	Hyperin	463.0885; 300.0271; 271.0244; 255.0294; 178.9983; 151.0036	(Li et al., 2022)
11	13.95	[M-H]^-^	479.0844	479.0831	C_21_H_20_O_13_	480.09	Cannabiscitrin	479.0836; 317.0299; 287.0197; 271.0238; 178.9983; 151.0034; 137.0242	(Qi et al., 2011)
12	14.22	[M-H]^-^	579.1399	579.1355	C_26_H_28_O_15_	580.14	Kaempferol 3-O-sambubioside	579.1390; 285.0325; 255.0302; 227.0356	(Guan et al., 2022)
13	14.49	[M-H]^-^	493.1002	493.0988	C_22_H_22_O_13_	494.11	Laricitrin 3-O-glucoside	493.1028; 330.0394; 315.0161; 287.0207; 271.0256; 151.0050	(Xiaozhou Jia et al., 2024 )
14	16.34	[M-H]^-^	755.2076	755.2040	C_33_H_40_O_20_	756.21	Complanatoside B	593.1544; 461.1112; 298.0483; 165.0202	(Xiaozhou Jia et al., 2024)
15	17.14	[M + FA-H]^-^	669.1724	669.1672	C_28_H_32_O_16_	624.17	Complanatuside	623.1641; 461.1102; 299.0568; 271.0626; 175.0200	(Xiaozhou Jia et al., 2024)
16	19.17	[M-H]^-^	841.2091	841.2044	C_36_H_42_O_23_	842.21	Complanatoside B + malonate	841.2141; 797.2235; 635.1674; 298.0498; 283.0267	(Xiaozhou Jia et al., 2024)
17	24.68	[M-H]^-^	593.1544	593.1512	C_27_H_30_O_15_	594.16	Tectorigenin 7-O-xylosylglucoside	593.1571; 298.0486; 283.0264; 255.0300	(Tang et al., 2016)
18	24.98	[M-H]^-^	931.2587	931.2514	C_43_H_48_O_23_	932.26	2-[4-(β-D-glucopyranosyloxy)phenyl]-5-hydroxy-3-[[2-O-[5-O-[3-(4-hydroxy-3-methoxyphenyl)-1-oxo-2-propenyl]-D-apio-β-D-furanosyl]-β-D-glucopyranosyl]oxy]-7-methoxy- 4H-1-Benzopyran-4-one	931.2570; 769.2018; 593.1521; 574.1423; 298.0496	(Guan et al., 2022)
19	26.37	[M + H]^+^	517.1337	517.1341	C_25_H_24_O_12_	516.13	Formononetin-7-O-β-D-glucoside-6″-O-malonate	517.1340; 269.0808; 254.0578; 237.0544; 213.0910	(Li et al., 2022)
20	27.77	[M-H]^-^	1017.2630	1017.2550	C_46_H_50_O_26_	1018.26	Complanatoside B + feruloyl + malonate	1017.2674; 973.2758; 811.2209; 635.1708; 617.1600; 298.0507; 193.0532	(Xiaozhou Jia et al., 2024)
21	28.34	[M-H]^-^	987.2496	987.2412	C_45_H_48_O_25_	988.25	Complanatoside B + coumaroyl + malonate	987.2515; 943.2623; 781.2060; 635.1684; 617.1568; 298.0492; 145.0300	(Xiaozhou Jia et al., 2024)
22	33.22	[M + H]^+^	1105.5815	1105.5789	C_54_H_88_O_23_	1104.57	Comploside II	1105.5841; 959.5248; 779.4576; 599.3944; 423.3615	(Kinjo et al., 1999)

### 3.2 Effects of ACF on body weight, liver function, inflammatory cytokines, and oxidative parameters in LF rats

The role of ACF in regulating body weight, liver function indicators, liver/spleen ratio, inflammatory cytokines, and oxidative parameters in CCl_4_-induced LF rats are exhibited in Figure 3. Relative to the control group, CCl_4_-induced LF rats exhibited a significantly accelerated decrease in body weight, consistent with the expected disease progression (*P* < 0.01). Conversely, ACF treatment significantly alleviated body weight loss in a dose-dependent fashion (*P* < 0.01) (Figure 3A). In comparison with normal rats, liver/spleen index showed a striking rise in the CCl_4_-induced LF rats (Figure 3B). ACF at 40 mg/kg markedly lowered the liver/spleen index (*P* < 0.05), while 80 mg/kg ACF specifically decreased the liver index in CCl_4_-induced LF rats (*P* < 0.05) (Figure 3B).

**FIGURE 3:**
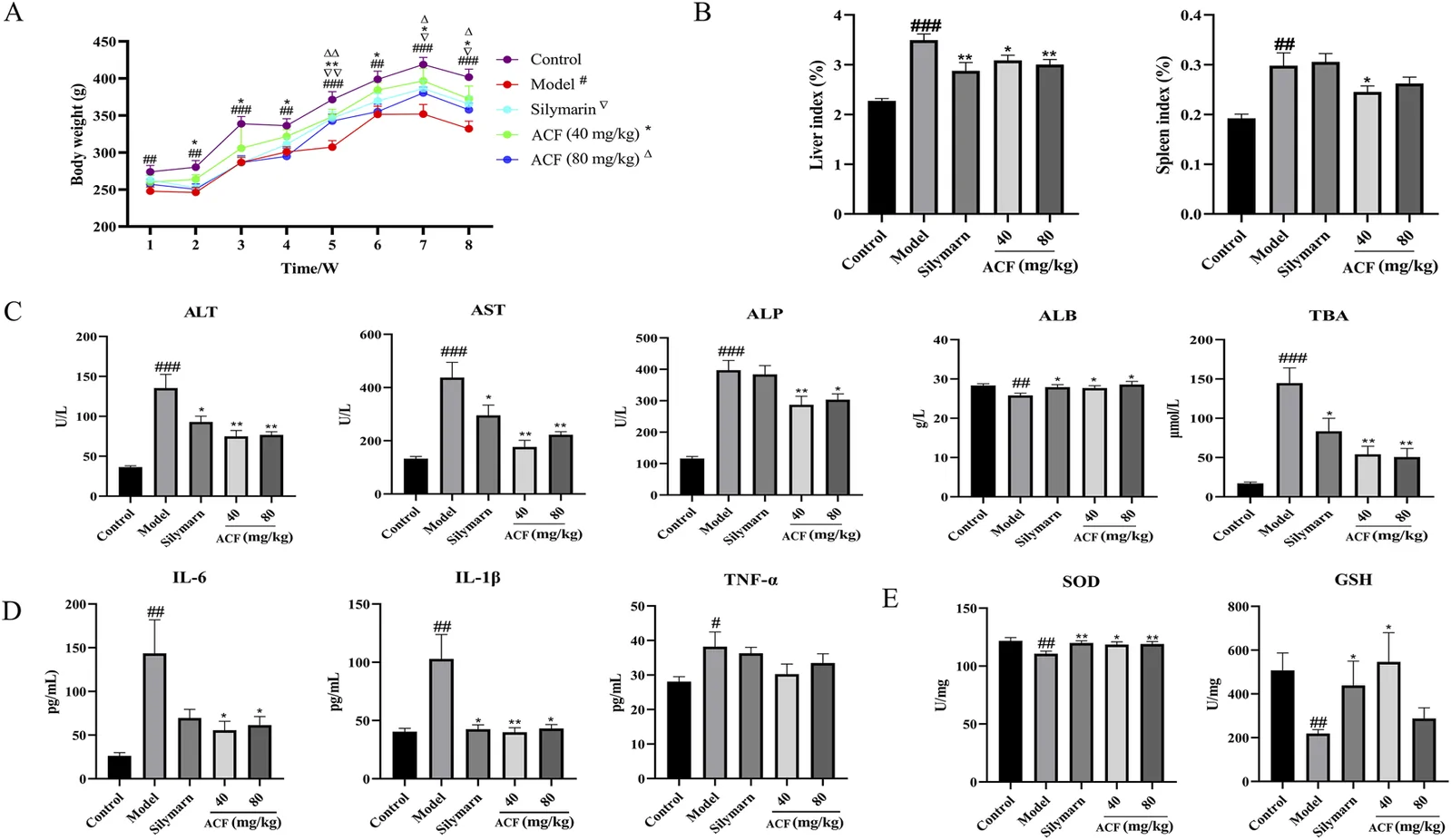
Effects of ACF on body weight, liver and spleen indices, liver function index, inflammatory factors and oxidation indices in LF rats (n = 6). **(A)** Body weight. **(B)** Liver and spleen indices. **(C)** Liver function index. **(D)** Inflammatory factors. **(E)** Oxidative stress markers. ^#^ *P* < 0.05, ^##^ *P* < 0.01, ^###^ *P* < 0.001, vs. the control group; ^*^ *P* < 0.05, ^**^ *P* < 0.01, ^***^ *P* < 0.001, ^△^ *P* < 0.05, ^△△^ *P* < 0.01, ^△△△^ *P* < 0.001, ^▽^ *P* < 0.05, ^▽▽^ *P* < 0.01, ^▽▽▽^ *P* < 0.001 vs. the model group.

As opposed to control rats, ALT, AST, ALP and TBA concentrations were markedly increased in LF model rats (*P* < 0.001), along with a notable decrease in ALB concentration (*P* < 0.01); as opposed to LF model rats, ACF treatment notably attenuated the alterations of these parameters (*P* < 0.05) (Figure 3C).

Model rats exhibited markedly elevated serum level of TNF-α, IL-6 and IL-1β in contrast to normal animals (*P* < 0.01); nevertheless, ACF notably attenuated these parameters in LF rats (*P* < 0.05) (Figure 3D). Hepatic SOD and GSH levels were dramatically lowered in LF rats versus controls (*P* < 0.05). Relative to model animals, ACF (40 mg/kg) dramatically reinstated both SOD and GSH levels (*P* < 0.05), while only SOD was notably increased in the 80 mg/kg group (*P* < 0.05) (Figure 3E). These findings demonstrate that ACF alleviates LF by exerting anti-inflammatory and antioxidant effects.

### 3.3 Serum fibrosis markers, hepatic histopathology, α-SMA immunostaining and collagen/TGF-β1 protein levels

Versus the normal rats, the serum concentration of these fibrotic biomarkers (collagen IV, HA, LN, PC-III and HYP) were obviously upregulated in model rats (*P* < 0.01). Conversely, ACF (40 and 80 mg/kg) treatment dramatically diminished all five fibrotic markers (*P* < 0.05) (Figure 4A).

**FIGURE 4:**
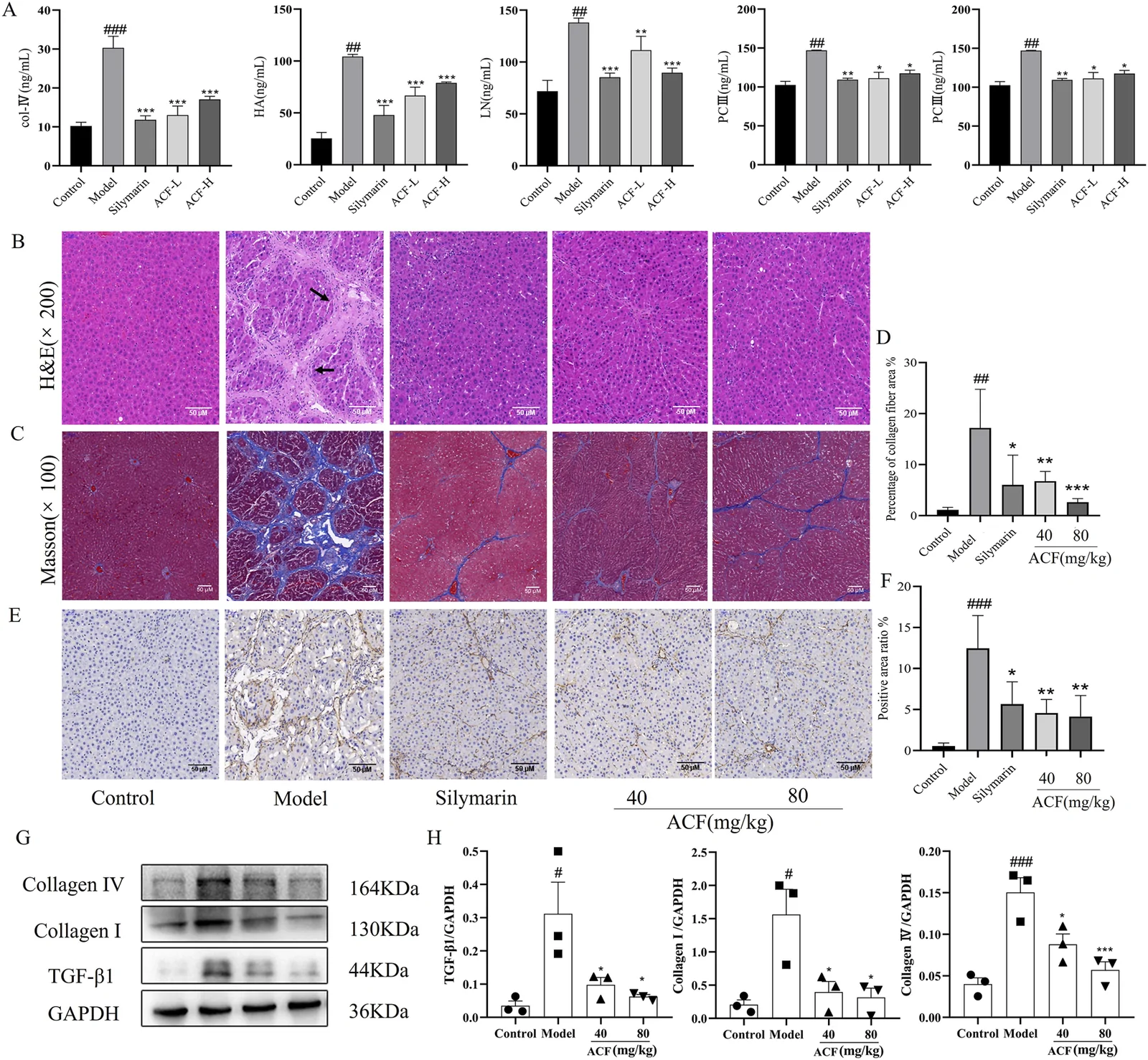
Effects of ACF on hepatic fibrosis in CCl_4_-treated LF rats. **(A)** Four indicators of LF and HYP content (n = 3). **(B)** HE staining image (200×). **(C)** Masson staining image (100×). **(D)** Collagen deposition in the liver (n = 3). **(E)** α-SMA immunohistochemical staining image (200×) and **(F)** ImageJ quantification of the immunohistochemically positive region (n = 3). **(G)** The protein expression levels of Collagen I, Collagen IV and TGF-β1 were probed by Western blotting and **(H)** quantified by Image Lab (n = 3). ^#^ *P* < 0.05, ^##^ *P* < 0.01, ^###^ *P* < 0.001, vs. the control group; ^*^ *P* < 0.05, ^**^ *P* < 0.01, ^***^ *P* < 0.001, vs. the model group.

Liver tissue from control rats displayed intact lobular architecture, orderly arranged hepatocytes, and clearly visible central vein walls. In contrast, liver tissue from CCl_4_-induced LF rats showed disordered hepatic cord arrangement, hepatocyte swelling and degeneration, central vein congestion, inflammatory cell infiltration, fibrous hyperplasia with cell deposition, focal hepatocyte necrosis, and disrupted hepatic cord structure. Notably, ACF treatment significantly ameliorated the structural damage in the liver tissue of LF rats (Figure 4B).

Normal rats showed only a few blue-stained collagen fiber areas. Compared with normal rats, CCl_4_-induced LF rats exhibited more collagen fibers, with a significantly enlarged blue-stained area (*P* < 0.001). Co-administration of silymarin (positive control) or ACF significantly reduced collagen fiber production and fibrosis, leading to a marked reduction in the blue-stained area in CCl_4_-induced LF rats (*P* < 0.05) (Figures 4C,D).

Normal rat livers showed only a few brown α-SMA-positive areas, with clearly visible hepatocyte morphology. CCl_4_-induced LF rats displayed a significantly increased and more widely distributed brown-stained area (*P* < 0.05) (Figure 4E). Conversely, compared with CCl_4_-induced LF rats, the ACF treatment groups showed a marked reduction in brown α-SMA-positive areas (*P* < 0.05) (Figure 4F).

CCl_4_-induced LF rats exhibited significantly upregulated protein levels of collagen I, collagen IV and TGF-β1 as opposed to control rats (*P* < 0.05). Importantly, ACF treatment (40 and 80 mg/kg) markedly inhibited collagen I, collagen IV and TGF-β1 levels in LF rats (*P* < 0.05) (Figures 4G,H).

### 3.4 Analysis of differentially expressed proteins

Relative to normal rats, 2,017 DEPs were screened out in CCl_4_-induced LF rats, with 1,333 upregulated and 684 downregulated (Figure 5A). Versus the model rats, 401 DEPs were detected following ACF administration, including 97 upregulated and 178 downregulated proteins (Figure 5B). In total, 264 DEPs were shared among the control, model and ACF groups (Figure 5C).

**FIGURE 5:**
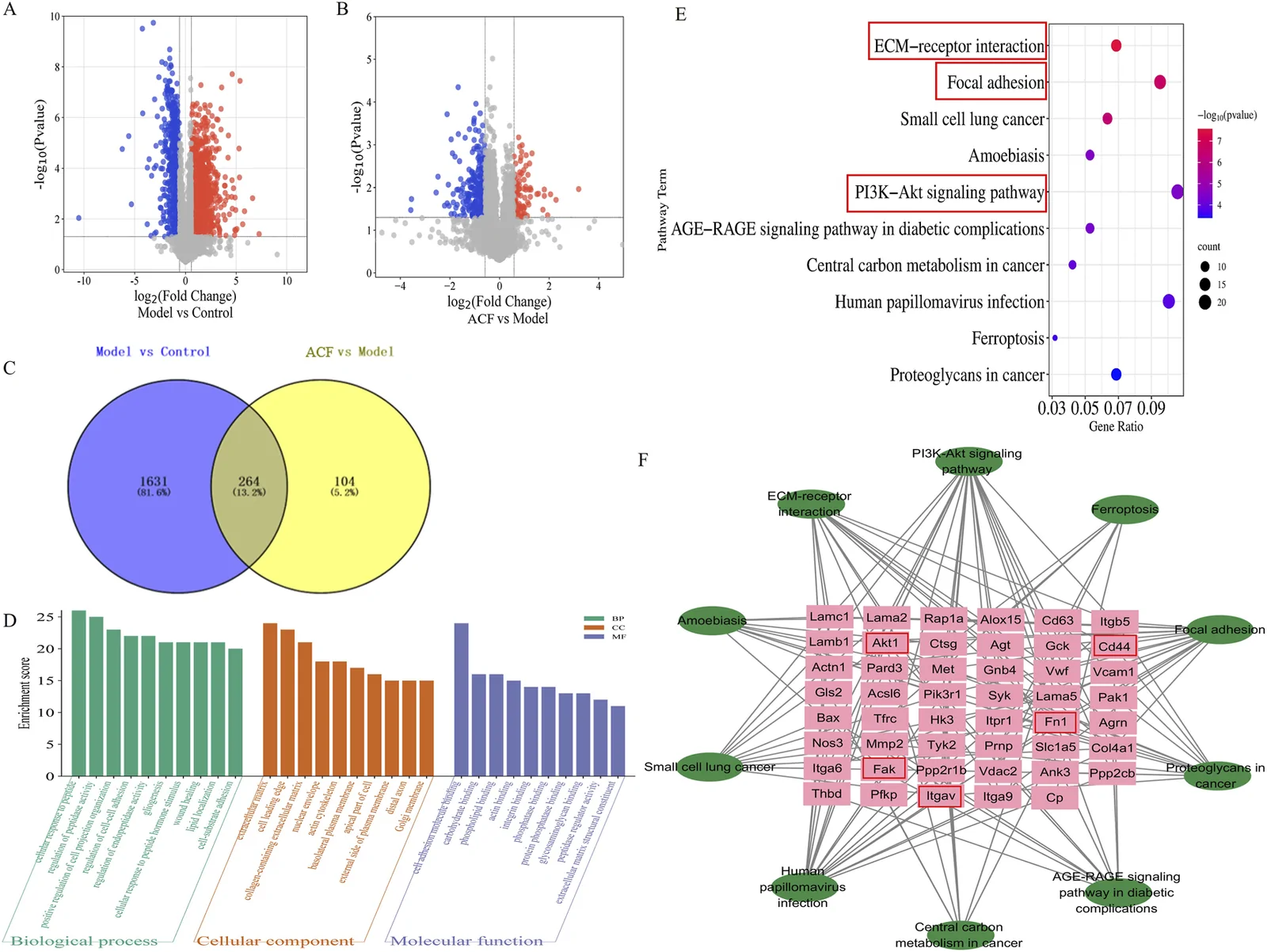
Proteomics analysis. **(A)** Volcano plot of the DEPs between the control and model groups. **(B)** Volcano plot of the DEPs between the ACF and model groups. **(C)** Venn diagram of the overlapping DEPs. **(D)** GO enrichment analysis of the ACF groups and model group. **(E)** KEGG pathway analysis of the ACF groups and model group. **(F)** PPI plot of the DEPs according to STRING network analysis.

Functional enrichment analysis demonstrated that the top enriched biological processes were cellular response to peptide, regulation of peptidase activity, and positive regulation of cell projection organization. The main cellular components were extracellular matrix (ECM), cell leading edge, and collagen-containing ECM. The primary molecular functions included cell adhesion molecule binding, carbohydrate binding, and phospholipid binding (Figure 5D). KEGG pathway analysis further revealed that DEPs were primarily concentrated in ECM–receptor interaction, focal adhesion (FA), and PI3K/AKT signaling pathways (Figure 5E).

Protein-protein interaction (PPI) network analysis evidenced that core proteins with high degree values included FN1, CD44, and ITGAV (derived from the ECM-receptor interaction pathway), FAK (derived from the FA pathway), and AKT1 (derived from the PI3K/AKT pathway) (Figure 5F), which are consistent with the KEGG pathway analysis.

The heatmap in Figure 6 shows differences in protein expression among groups, with blue indicating downregulation and red indicating upregulation. The model group displayed more downregulated proteins (blue bands), including key proteins such as AKT1, FN1, FAK, CD44, and ITGAV, whereas these key proteins were upregulated (red bands) in the ACF group.

**FIGURE 6:**
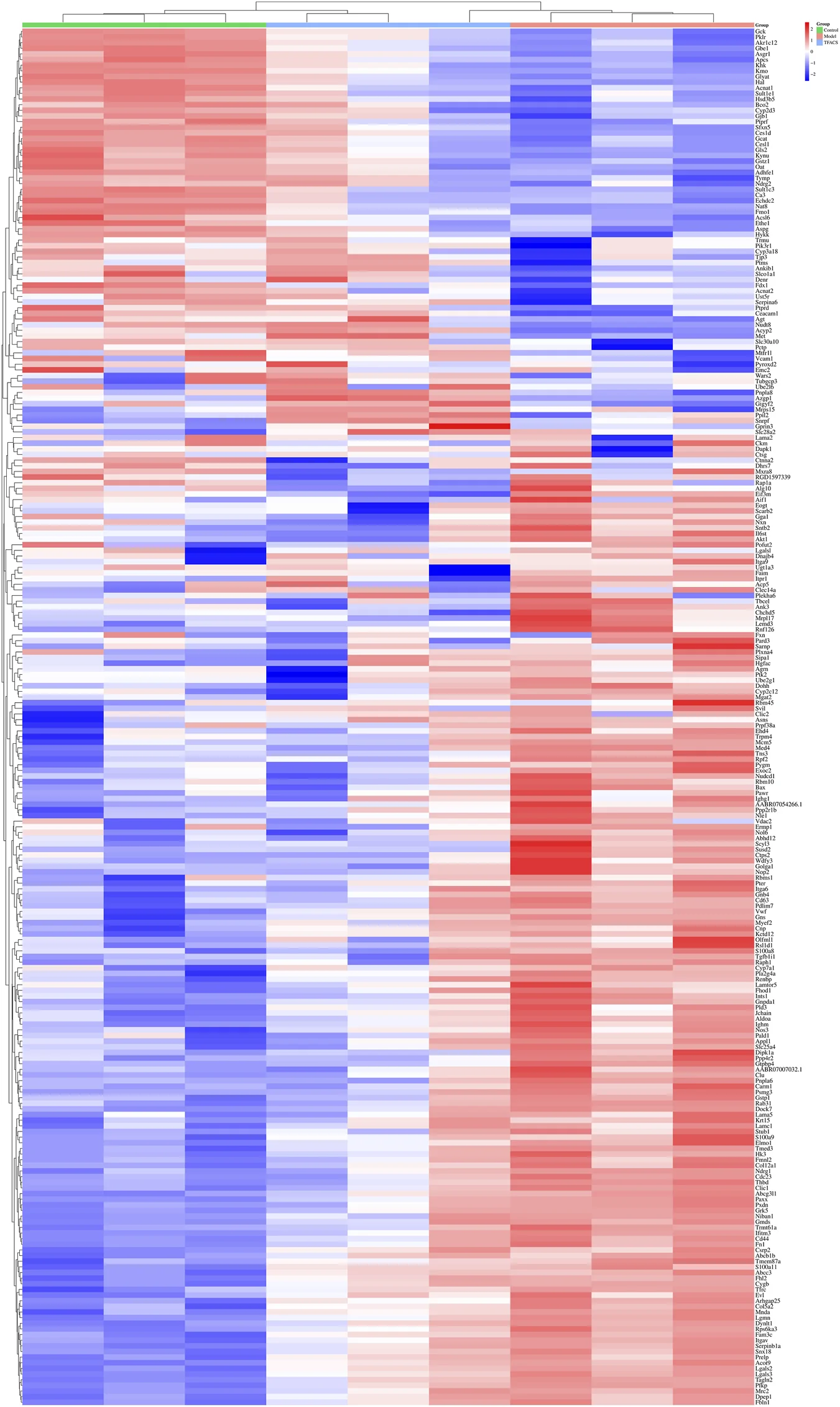
Cluster analysis of the differentially expressed proteins among the three groups. The colours represent changes in protein expression: blue indicates a negative correlation, and red indicates a positive correlation; the deeper the colour is, the stronger the correlation.

### 3.5 Validation of key Anti-LF targets of ACF at gene and protein levels

The mRNA levels of AKT1, FN1, FAK, CD44 and ITGAV were significantly upregulated in CCl_4_-induced LF rats relative to controls (*P* < 0.05). ACF (40 mg/kg) treatment exerted a notable inhibitory effect on these gene expressions (*P* < 0.05) (Figure 7A).

**FIGURE 7:**
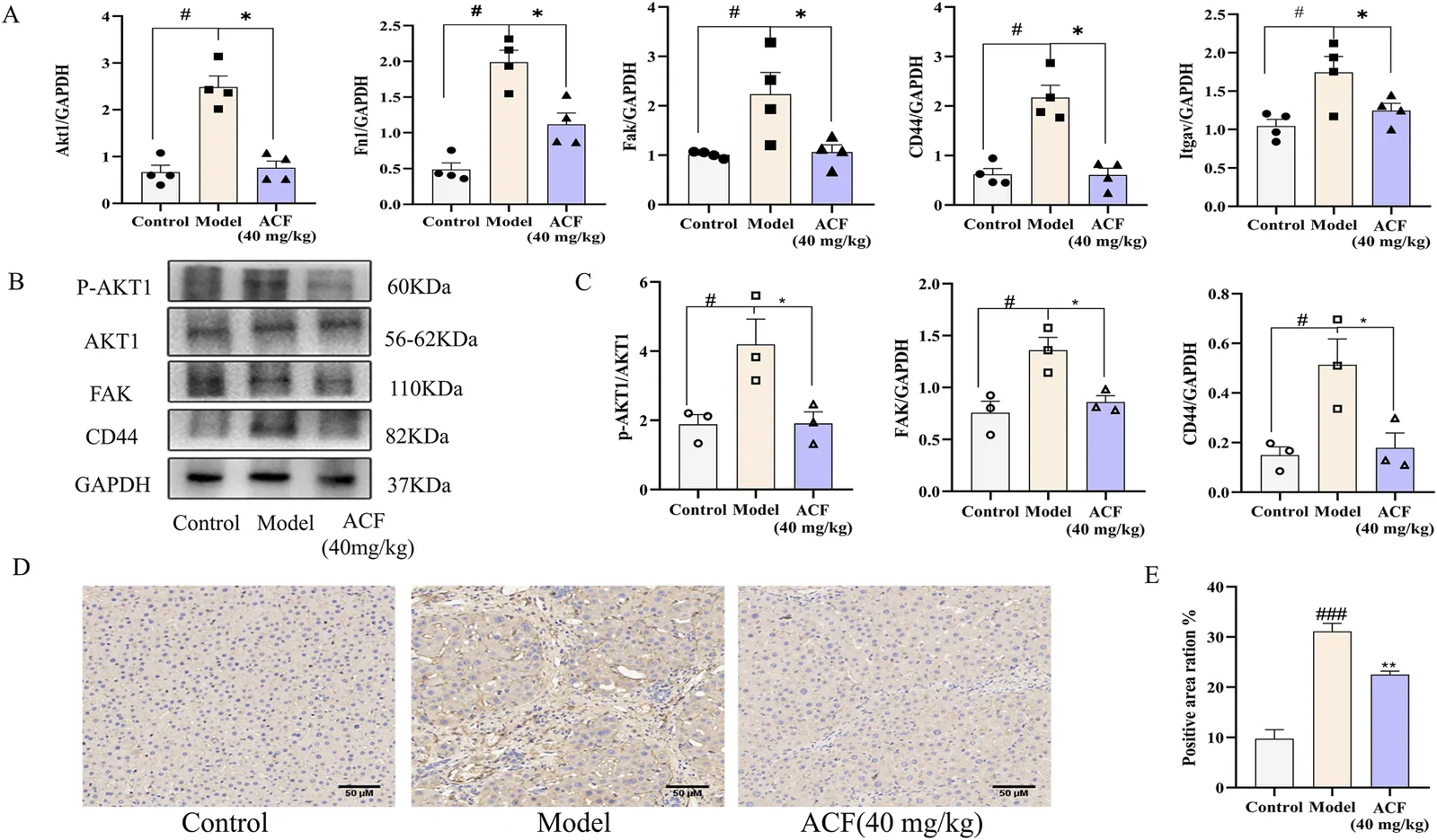
Validation of key ACF targets against LF at the gene and protein levels (n = 6). **(A)** The gene expression levels of AKT1, FAK, ITGAV, FN1 and CD44 were probed by qRT-PCR. **(B)** The protein expression levels of AKT1, p-AKT1, FAK and CD44 were probed by Western blotting and **(C)** quantified by Image Lab. **(D)** FN1 immunohistochemical staining images (200×) and **(E)** area of the immunohistochemically positive region measured by ImageJ. ^#^ *P* < 0.05, ^##^ *P* < 0.01, ^###^ *P* < 0.001, vs. the control group; ^*^ *P* < 0.05, ^**^ *P* < 0.01, ^***^ *P* < 0.001, vs. the model group.

Similarly, protein level of AKT1, p-AKT1, FAK and CD44 were significantly elevated in LF model rats relative to controls (*P* < 0.05). ACF (40 mg/kg) administration notably attenuated the expression of these target proteins (*P* < 0.05) (Figures 7B,C).

FN1 immunoreactivity was sparse in normal rat livers, whereas FN1 staining was markedly enhanced in CCl_4_-induced fibrotic livers (*P* < 0.05). ACF (40 mg/kg) markedly lowered the proportion of brown FN1-positive staining in CCl_4_-induced LF rats (*P* < 0.05) (Figures 7D,E).

### 3.6 ACF inhibits hepatic stellate cell activation *in vitro*

To elucidate the mechanism by which ACF protects the liver from fibrotic injury, *in vitro* experiments were conducted using HSC-T6 cells. ACF exhibited no cytotoxicity to HSC-T6 cells (Figure 8A). To date, TGF-β1 represents the principal profibrotic factor characterized (Xu et al., 2016). After treatment with TGF-β1 (10 ng/mL), HSC-T6 cell viability was significantly increased (*P* < 0.05). However, ACF (0.1–1 μg/mL) distinctly decreased the viability of TGF-β1-activated HSC-T6 cells (Figure 8B).

**FIGURE 8:**
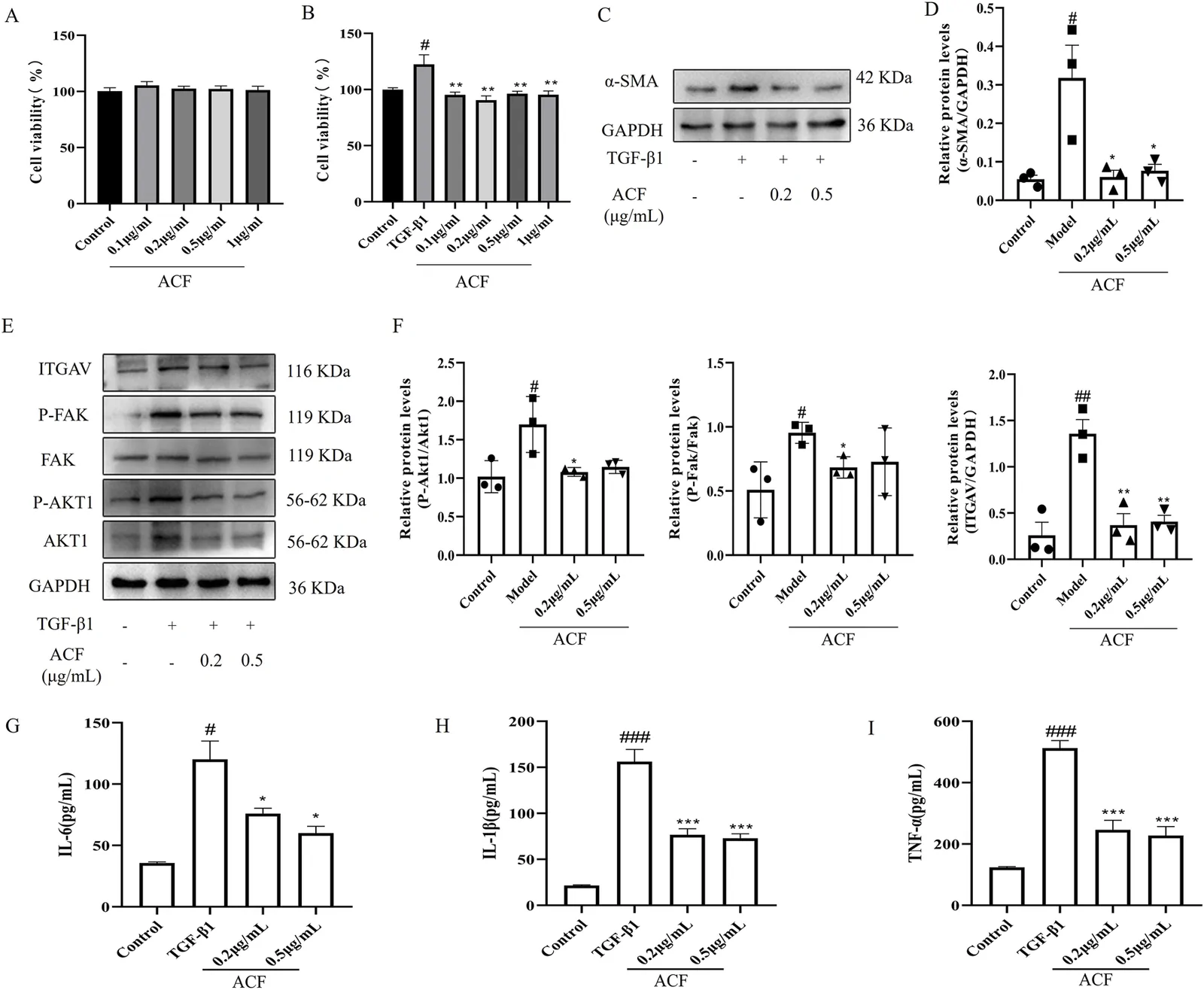
Effect of ACF on HSC activation. **(A)** The effect of ACF on the viability of HSCs was detected by a CCK-8 assay (n = 3). **(B)** The effect of ACF on the viability of activated HSCs was detected by a CCK-8 assay (n = 3). **(C)** The protein expression of α-SMA was probed by Western blotting and **(D)** quantified by Image Lab (n = 3). **(E)** The protein expression levels of AKT1, p-AKT1, FAK, p-FAK and ITGAV were probed by Western blotting and **(F)** quantified by Image Lab. Effects of ACF on the levels of IL-6 **(G)**, IL-1β **(H)** and TNF-α **(I)** in HSC-T6 cells (n = 6). ^#^ *P* < 0.05, ^##^ *P* < 0.01, ^###^ *P* < 0.001, vs. the control group; ^*^ *P* < 0.05, ^**^ *P* < 0.01, ^***^ *P* < 0.001, vs. the model group.

Prior research has demonstrated that ACF (0.1–0.5 μg/mL) is non-toxic to L0-2 cells (Chen et al., 2024). Combined with our CCK-8 results showing no obvious cytotoxicity in unstimulated HSC-T6 cells and inhibitory effects on TGF-β1-activated HSC-T6 cells. Therefore, ACF at 0.2 and 0.5 μg/mL were selected for subsequent experiments. TGF-β1 exposure markedly elevated α-SMA protein level in HSC-T6 cells relative to controls, while ACF (0.2 or 0.5 μg/mL) significantly suppressed α-SMA levels in TGF-β1-stimulated cells, suggesting antifibrotic effects via inhibiting HSC activation (Figures 8C,D).

Relative to the control group, TGF-β1 stimulation markedly raised ITGAV protein expression as well as the p-AKT1/AKT1 and p-FAK/FAK ratios in HSC-T6 cells. Furthermore, ACF (0.2 or 0.5 μg/mL) markedly downregulated ITGAV protein expression and decreased the p-AKT1/AKT1 and p-FAK/FAK ratios in activated HSC-T6 cells (Figures 8E,F), validating at the cellular level the proteomics-identified anti-LF targets of ACF.

Activated HSC-T6 cells exhibited notably higher IL-6, IL-1β and TNF-α levels than the control group (*P* < 0.05). ACF (0.2 or 0.5 μg/mL) markedly suppressed these pro-inflammatory cytokines (*P* < 0.05) (Figures 8G–I). These findings imply that ACF exerts anti-inflammatory actions in activated HSC-T6 cells.

### 3.7 ACF inhibits HSC activation by suppressing the FAK/AKT signaling pathway

#### 3.7.1 Effects of ACF and the AKT inhibitor MK2206 on HSC activation

FAK and AKT serve as key molecules in the FA and PI3K/AKT pathways, respectively. As presented in Figures 9A,B, TGF-β1 stimulation notably increased α-SMA expression and p-FAK/FAK ratio in HSC-T6 cells relative to controls. Conversely, ACF (0.2 or 0.5 μg/mL) or the AKT inhibitor MK2206 (1 μM) markedly decreased α-SMA levels and p-AKT1/AKT1 ratio in TGF-β1-treated cells, suggesting that ACF inhibits HSC activation by suppressing AKT1 phosphorylation. Treatment with ACF (0.2 or 0.5 μg/mL) or MK2206 (1 μM) induced a significant decline in the concentrations of IL-6, IL-1β, and TNF-α (*P* < 0.05; Figures 9C–E).

**FIGURE 9:**
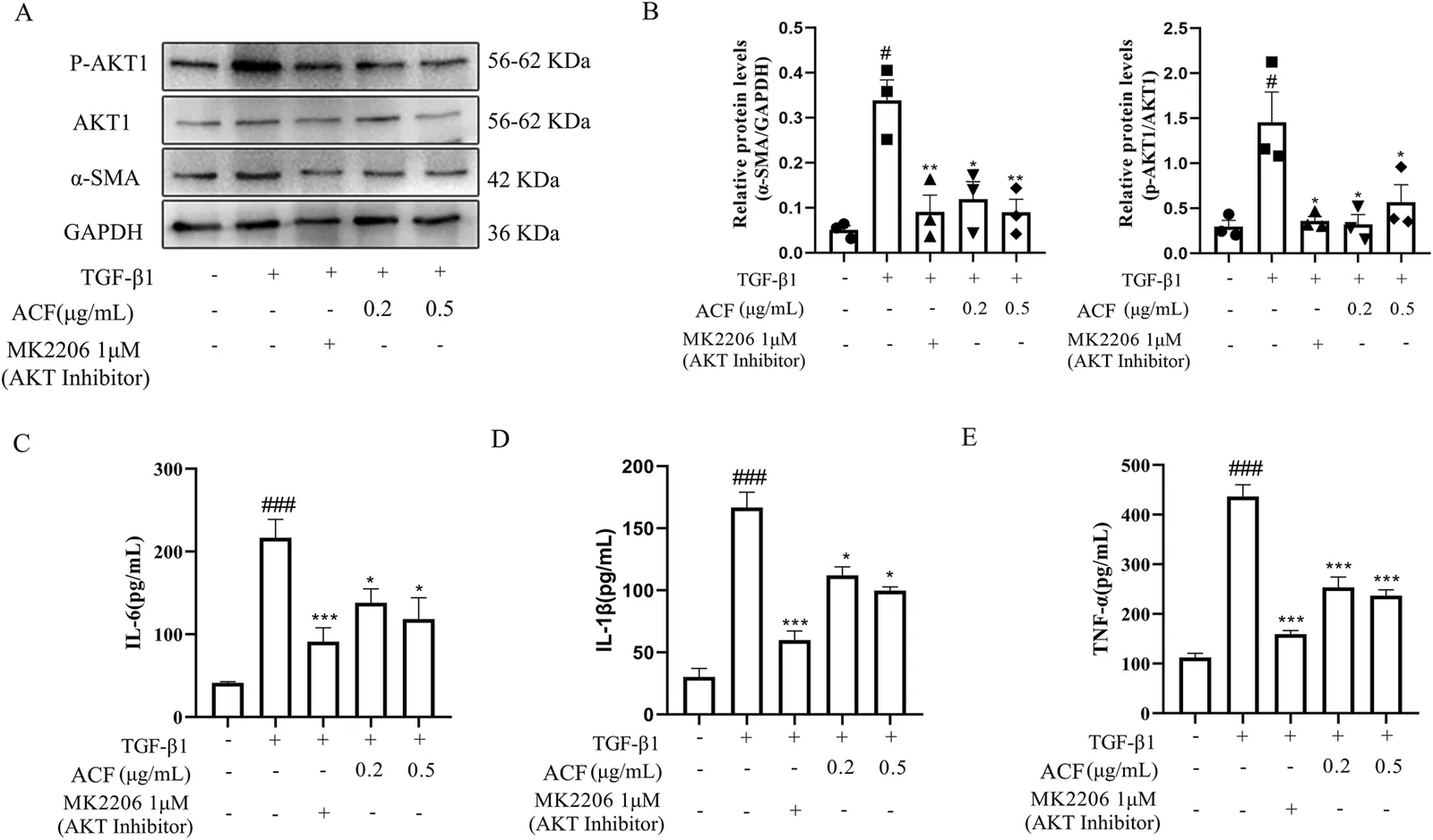
Effects of ACF on the FAK/AKT signaling pathway in HSCs. **(A)** HSC-T6 cells were treated with 0.5 μg/mL ACF and/or the AKT phosphorylation inhibitor MK2206 (1 μM) and 10 ng/mL TGF-β for 24 h **(B)** AKT1, p-AKT1 and α-SMA levels were detected and quantified by Image Lab. Effects of ACF on the levels of IL-6 (n = 3) **(C)**, IL-1β **(D)** and TNF-α **(E)** in HSC-T6 cells (n = 6). ^#^ *P* < 0.05, ^##^ *P* < 0.01, ^###^ *P* < 0.001, vs. the control group; ^*^ *P* < 0.05, ^**^ *P* < 0.01, ^***^ *P* < 0.001, vs. the model group.

#### 3.7.2 Effects of ACF and the FAK inhibitor PF-562271 on HSC activation

Compared with control cells, TGF-β1-exposed HSC-T6 cells displayed robust increases in α-SMA protein and in the ratios of p-AKT1/AKT1 and p-FAK/FAK (*P* < 0.05). These TGF-β1-induced changes were effectively counteracted by either ACF (0.2 or 0.5 μg/mL) or the FAK inhibitor PF-562271 (1 μM), which significantly lowered α-SMA and the two phosphorylation ratios (*P* < 0.05; Figures 10A,B).

**FIGURE 10:**
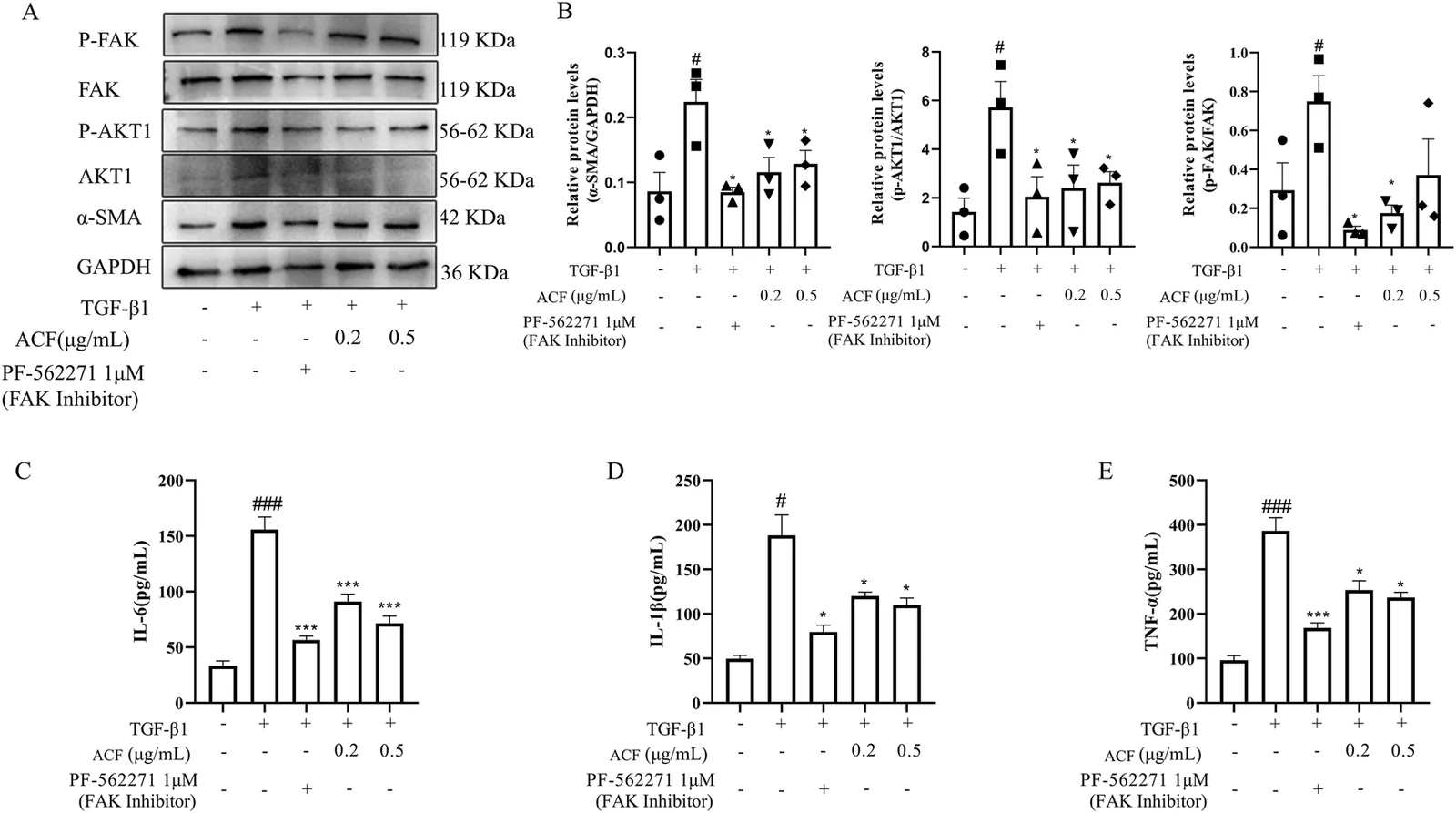
Effects of ACF on the FAK/AKT signaling pathway in HSCs. **(A)** HSC-T6 cells were treated with 0.5 μg/mL ACF and/or the FAK phosphorylation inhibitor PF-562271 (1 μM) and 10 ng/mL TGF-β for 24 h **(B)** AKT1, p-AKT1, p-FAK, FAK and α-SMA levels were detected and quantified by Image Lab. Effects of ACF on the levels of IL-6 (n = 3) **(C)**, IL-1β **(D)** and TNF-α **(E)** in HSC-T6 cells (n = 6). ^#^ *P* < 0.05, ^##^ *P* < 0.01, ^###^ *P* < 0.001, vs. the control group; ^*^ *P* < 0.05, ^**^ *P* < 0.01, ^***^ *P* < 0.001, vs. the model group.

Treatment with ACF (0.2 or 0.5 μg/mL) or PF-562271 (1 μM) effectively suppressed the level of IL-6, IL-1β, and TNF-α (*P* < 0.05; Figures 10C–E). These results confirm that ACF reduces inflammation through inhibition of the FAK/AKT pathway, thereby suppressing HSC activation.

### 3.8 Analysis of differential metabolites

Metabolomics data were processed by PLS-DA to visualize differences in metabolite composition among samples (Figure 11). The three groups (Control, Model, and ACF) exhibited a clear clustering separation trend (Figure 11A), with good intra-group aggregation, indicating high experimental reproducibility and data stability. Differential metabolites between groups were visualized using volcano plots. Relative to the control group, 102 DM were identified in CCl_4_-induced LF rats, with 54 increased and 48 decreased (Figure 11B). Versus the model group, 108 DM were detected in ACF-treated rats, including 61 upregulated and 47 downregulated (Figure 11C). A Venn diagram revealed 86 shared DMs across the three groups (Figure 11D).

**FIGURE 11:**
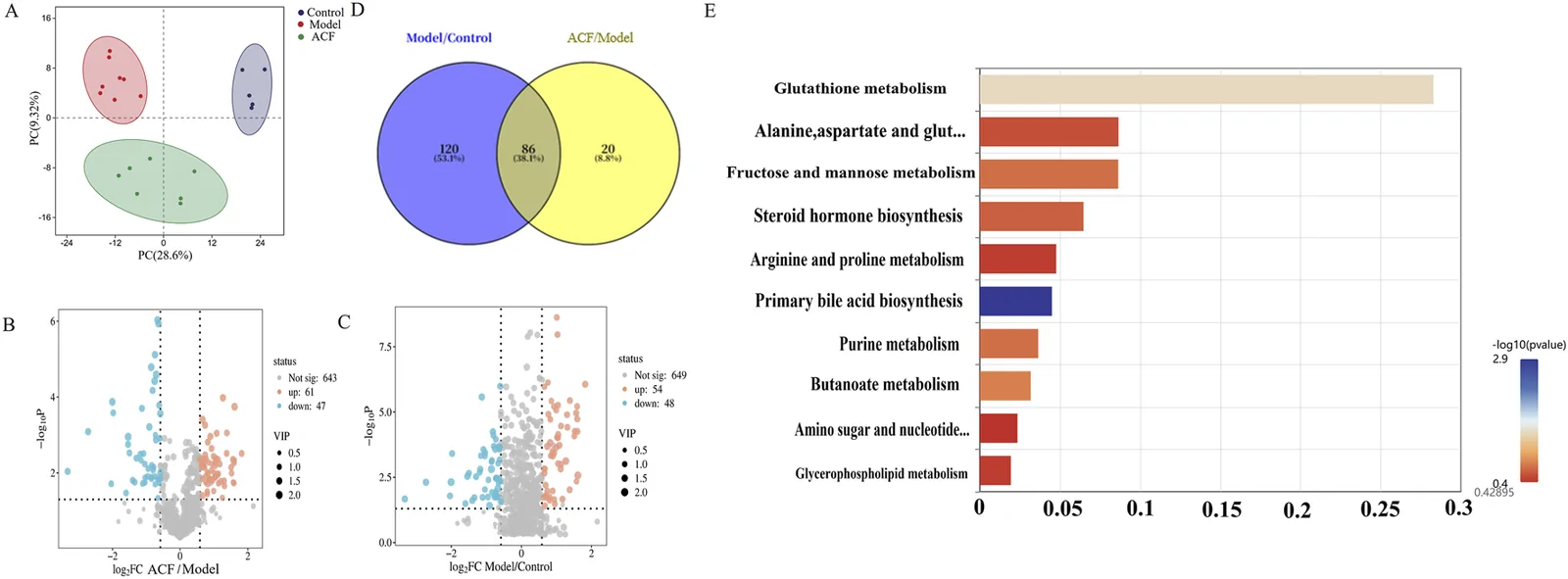
Metabolomics analysis. **(A)** PLS-DA analysis of differential samples. **(B)** Volcano plot of differential metabolites between ACF and model groups. **(C)** Volcano plot of differential metabolites between model and ACF groups. **(D)** Venn diagram of overlapping differential metabolites. **(E)** KEGG pathway analysis of differential metabolites between ACF and model groups.

Pathway enrichment analysis indicated that these DMs were primarily enriched in glutathione metabolism, alanine, aspartate and glutamate metabolism, and fructose and mannose metabolism (Figure 11E).

### 3.9 Integrative analysis of differential proteins and differential metabolites

Integrating the analyses of DEPs and DMs, KEGG pathway enrichment analysis revealed a total of 48 pathways (*P* < 0.05) (Figure 12). The top 20 pathways were selected for correlation analysis, including ECM-receptor interaction, fructose and mannose metabolism, PI3K/AKT signaling pathway, and primary bile acid biosynthesis, involving a total of 40 proteins and 6 metabolites.

**FIGURE 12:**
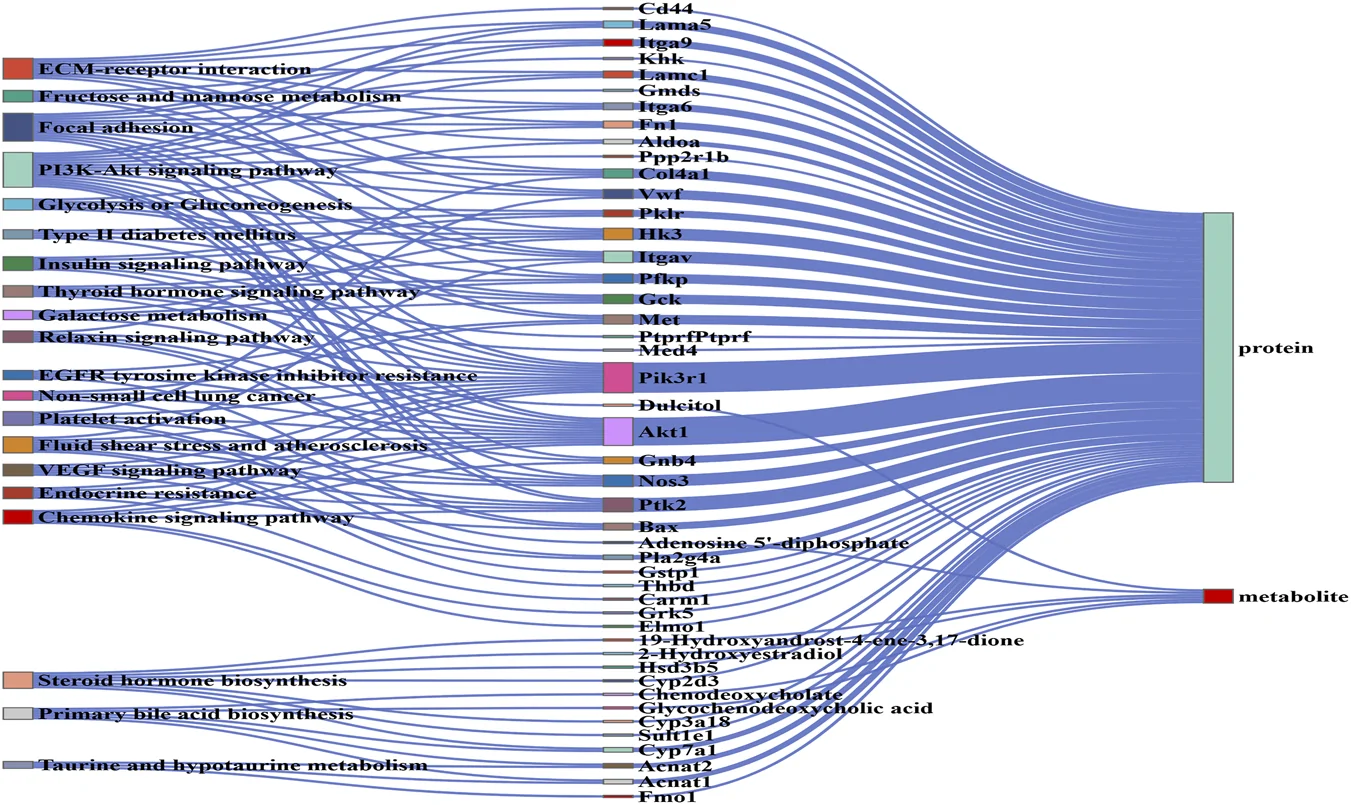
Sankey diagram of integrated KEGG pathway analysis for proteins and metabolites.

Correlation analysis between the two omics datasets showed significant positive or negative correlations among the various proteins and metabolites (Pearson correlation coefficient cut-off set at ≥ 0.7 or ≤ −0.7) (Figure 13A). Notably, glycochenodeoxycholic acid (GCDCA) was positively correlated with GSTP1*,* LAMC1*,* CD44*,* HK3*,* VWF*,* GMDS*,* and ITGA9 (*P* < 0.05), and negatively correlated with CYP3A18 (*P* < 0.05). Dulcitol was positively associated with KHK*,* ACNAT2*,* SULT1E1*,* GCK*,* CYP3A18*,* ACNAT1*,* HSD3B5*,* PKLR*,* FMO1, and CYP2D3 (*P* < 0.05), while negatively correlated with FN1*,* ITGAV*,* GRK5*,* CD44*,* PFKP*,* VWF*,* and THBD (*P* < 0.05). Chenodeoxycholate exhibited positive correlations with GSTP1*,* LAMC1*,* CD44*,* HK3*,* VWF*,* and GMDS (*P* < 0.05) and negative correlations with PTPRF*,* GCK*,* and CYP3A18 (*P* < 0.05). Adenosine 5′-diphosphate was positively linked to ACNAT2*,* CYP3A18*,* and HSD3B5 (*P* < 0.05) and negatively related to FN1*,* MED4*,* ITGAV*,* GRK5*,* AKT1*,* PFKP*,* and THBD (*P* < 0.05). 2-Hydroxyestradiol was negatively correlated with PTPRF and PIK3R1 (*P* < 0.05). 19-Hydroxyandrost-4-ene-3,17-dione were positively associated with GSTP1 (*P* < 0.05) and an inverse correlation with PTPRF and PIK3R1 (*P* < 0.05) (Figure 13B).

**FIGURE 13:**
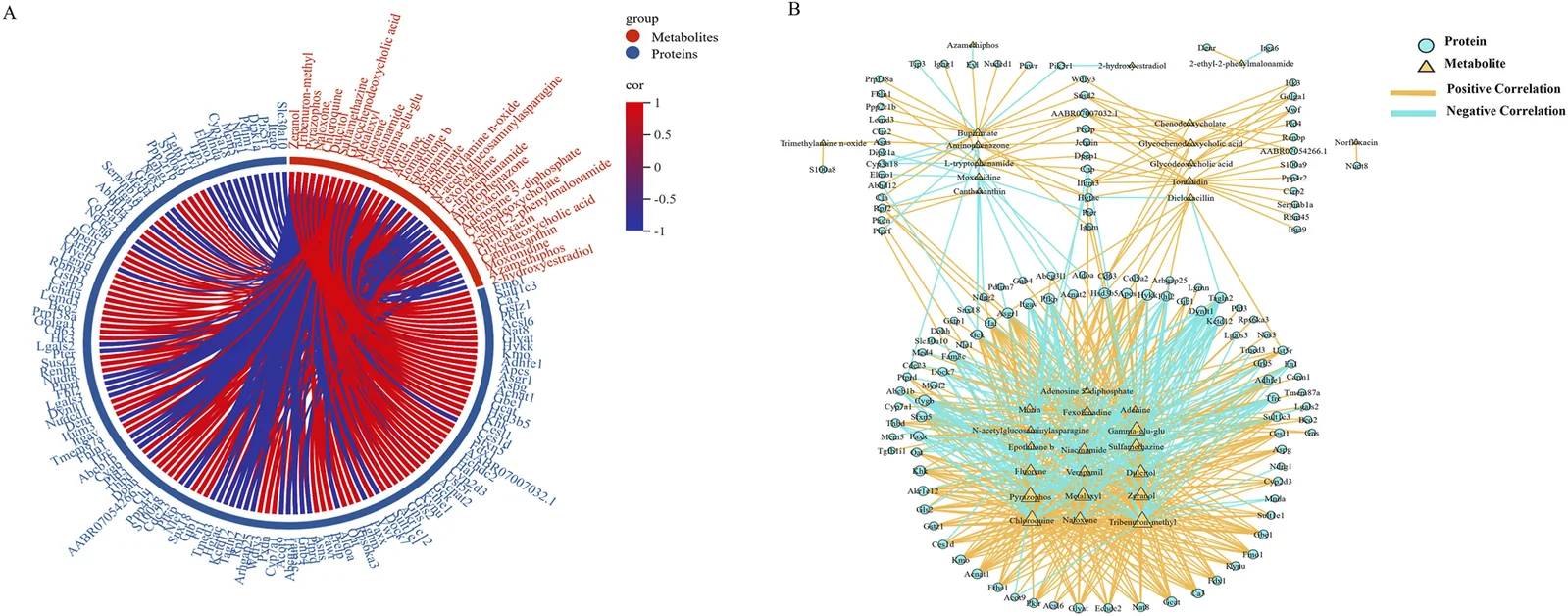
Integrated analysis of correlations between proteomics and non-targeted metabolomics data. **(A)** Correlation cycle diagram between proteomics and metabolomics. **(B)** Pearson correlations between proteomics and non-targeted metabolomics data. Pearson coefficients were filtered at a threshold of ≥0.7 or ≤ −0.7.

**FIGURE 14:**
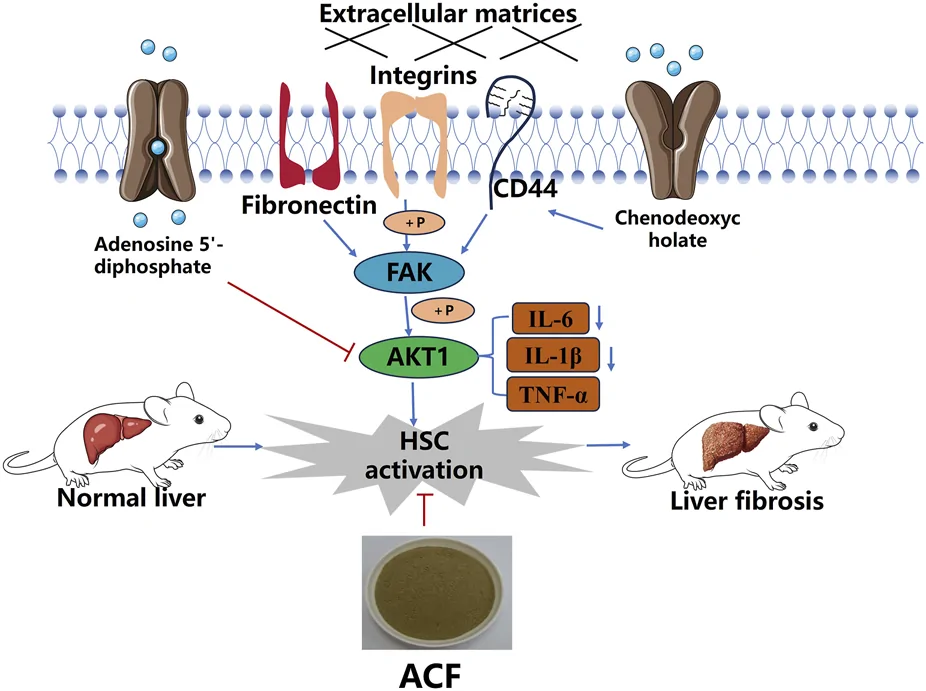
Pathways modulated by ACF in CCl_4_-induced LF rats.

## 4 Discussion

ACF is a total flavonoid extract derived from ACS. Our prior investigations have verified that ACF exhibits hepatoprotective activity against chronic liver injury (Chen et al., 2024). Extensive studies have demonstrated that LF-associated symptoms are improved by flavonoids (Miao et al., 2019; Li et al., 2021). Hence, we propose the hypothesis that ACF may exert a protective effect against LF.

*In vitro* combined with *in vivo* experiments further validated the anti-LF capacity of ACF. ACF substantially reduced the degree of fibrosis in the model rats, diminished hepatocyte edema, necrosis, and inflammatory cell infiltration, alleviated liver dysfunction, and suppressed α-SMA expression in the livers of the model rats. Further *in vitro* experiments indicated that ACF notably reduced the viability of HSCs and substantially decreased α-SMA protein expression in TGF-β1-activated HSC-T6 cells. The data suggest that ACF inhibits the HSC activation, thereby improving LF.

During the progression of LF, numerous protein molecules undergo remarkable changes (Zardi et al., 2008). Proteomics analysis can efficiently identify disease-associated DEPs, therapeutic targets, and action pathways (Xin et al., 2018). Our proteomics data reveal that ECM, FA, and the PI3K/AKT pathway were engaged in the antifibrotic process of ACF. Further PPI analysis indicated that proteins such as CD44, ITGAV, FN1, FAK, and AKT1 were critical targets within these three signaling pathways (Figure 5).

Evidence indicates that the ECM pathway is a vital part of the FA and PI3K/AKT signaling pathways (Chen et al., 2017). Adhesion of cells to the extracellular matrix is mainly mediated by ECM receptors, with integrins, fibronectin, elastin, and proteoglycans playing particularly important roles. As a member of the integrin family, ITGAV activates the FA signaling pathway, thereby stimulating pathways such as PI3K/AKT (Mitra et al., 2005). FN1 is primarily produced by activated HSC and is an ECM protein (Moretti et al., 2022). Under the regulation of various signaling pathways, it modulates cell adhesion, migration, and proliferation, driving the pathogenesis and progressive aggravation of fibrosis (Dalton and Lemmon, 2021). Alterations in CD44 protein expression or function are associated with various pathological states. In the liver, HSC are one of the primary cell types expressing CD44, and its expression increases with the progression of fibrosis (Ponta et al., 2003). ACF exerts an anti-fibrotic effect by inhibiting ECM pathways, specifically through downregulation of integrin, CD44, and FN1 expression. Significant upregulation of ITGAV, CD44, and FN1 (mRNA and/or protein) was observed in LF rats relative to control rats, while ACF treatment led to a marked reduction in their expression (Figure 7). These observations imply that the ECM pathway may contribute crucially to the anti-LF effects of ACF.

FA is crucial for HSC activation, and blockade of this signaling cascade contributes to LF reversal (Kumar et al., 2014; Wang et al., 2017). As a cytoplasmic tyrosine kinase involved in FA signaling, FAK upregulation further facilitates HSC activation (Zhao et al., 2017). We assessed FAK mRNA and protein expression in LF rats and HSC-T6 cells. ACF treatment group significantly reduced FAK gene and protein expression in LF model rats (Figures 7A–C). Furthermore, in TGF-β1-induced HSC-T6 cells, FAK phosphorylation was enhanced and accompanied by upregulation of α-SMA protein expression; ACF treatment reversed these changes (Figures 10C–F). The data imply that FAK, as a key molecule in the FA signaling, is the core mediator of ACF against LF.

Activated by Y397 site phosphorylation through integrin-mediated signaling, FAK triggers the PI3K survival signaling cascade, induces AKT phosphorylation, and ultimately promotes HSC activation (Bao et al., 2019). This suggests that FAK may participate in HSC activation by regulating the PI3K/AKT pathway. This signaling axis is intrinsically linked to multiple biological processes, including inflammatory injury, parenchymal cell apoptosis, and the proliferation of mesenchymal cells (particularly fibroblasts) (El Nashar et al., 2021). As a key kinase regulating apoptosis in the PI3K/AKT pathway, the activation status of AKT1 in HSC can influence cell proliferation and apoptosis, thereby promoting the development of LF (Paik et al., 2009). Studies have shown that AKT1, as a key hub target, mediates the therapeutic effects of traditional bioactive compounds and flavonoids on chronic hepatic metabolic disorders and inflammation (Ren et al., 2024). Based on this, we examined the phosphorylation levels of AKT1 in LF model rats and HSC-T6 cells. In the study, the LF model group exhibited markedly elevated AKT1 phosphorylation levels compared with the normal rats; however, following ACF treatment, these levels were significantly reduced (Figures 7A–C). In TGF-β1-induced HSC-T6 cells, both AKT1 phosphorylation levels and α-SMA expression were upregulated; ACF treatment reversed these changes (Figures 8C–F). These results indicate that AKT1 in the PI3K/AKT pathway may exert a pivotal effect on the anti-LF effects of ACF.

To test the effect of FAK/AKT1 signaling in ACF against LF, we treated TGF-β1-activated HSC-T6 cells with either the AKT1 inhibitor (MK2206) or the FAK inhibitor (PF-562271) alone, or in combination with ACF. MK2206 is an allosteric AKT inhibitor, and it effectively inhibits AKT activity when used alone (Hirai et al., 2010; Pal et al., 2010). PF-562271 inhibits FAK activity by targeting phosphorylation at the FAK Y397 site, thereby blocking downstream signaling cascades and AKT phosphorylation, and consequently suppressing HSC activation (Yang et al., 2024). Recent studies have shown that pharmacological inhibition of FAK using PF-562271 can attenuate TGF-β1-induced HSC activation and suppress aerobic glycolysis via the FAK/cyclin D1/c-Myc/MCT-1 axis, further supporting the functional link between FAK signaling and metabolic reprogramming during liver fibrosis (Huang et al., 2025). Experimental results showed that both ACF and MK2206 could reverse AKT1 phosphorylation and reduce the protein expression of α-SMA (Figure 9). Similarly, both ACF and PF-562271 could reverse TGF-β1-induced FAK and AKT1 phosphorylation and reduce α-SMA level (Figure 10). These results confirm that ACF exerts its antifibrotic effect by inhibiting HSC activation through the blockade of the FAK/AKT1 signaling cascade. However, although pharmacological inhibition experiments support the involvement of the FAK/AKT signaling pathway, this study did not include a control group treated with the inhibitor alone. This limits our ability to distinguish between the independent effects of pathway inhibition and the synergistic effects between the inhibitor and ACF, and further research is needed.

Metabolomic analysis provided additional validation for how ACF exerts its effects at the metabolic level. PLS-DA analysis revealed clear cluster separation among samples, indicating that the model successfully induced metabolic dysregulation and that ACF intervention significantly reversed this abnormality. DMs screening revealed 102 DM between the normal and LF rats, and 108 DM between the LF and ACF-treated rats. Among these, 86 overlapping DMs suggest that ACF primarily exerts its effects by improving the model-induced metabolic dysregulation. Glutathione metabolism—the most influential pathway in metabolomics—corresponds with the anti-inflammatory and antioxidant mechanisms revealed by proteomics. ACF not only directly enhances the body’s antioxidant capacity by upregulating SOD activity and GSH levels but also mitigates oxidative damage via the regulation of metabolites associated with the glutathione metabolism pathway. This effect synergizes with the cell-protective action mediated by the FAK/AKT pathway, as revealed by proteomics. Furthermore, primary bile acid biosynthesis is the most prominent pathway, suggesting that ACF may have a potential regulatory effect on the gut microbiome and lipid metabolism (Zhuang et al., 2024). The enrichment of amino acid and glucose metabolism pathways indicates that ACF can alleviate energy metabolism disorders during the progression of LF by improving energy metabolism efficiency; this process may be closely related to cellular metabolic reprogramming regulated by the ECM receptor interaction pathway identified in proteomics (Chen et al., 2023; Gao, 2024).

Multi-omics analysis further identified 48 protein-metabolic synergistic regulatory pathways, revealing significant correlations (Pearson correlation coefficients ≥0.7 or ≤ −0.7) between 40 differentially expressed proteins and 6 key differentially expressed metabolites. Based on these findings, we established a comprehensive mechanism spanning from molecular targets to metabolic phenotypes, elucidating the molecular logic by which ACF exerts its fibrosis-repressing actions by regulating core protein expression and metabolic pathways, thereby validating its dual protein-metabolic regulatory model (Majtan et al., 2024). Correlation analysis results showed that a marked positive/negative correlation was observed between DMs and core target proteins, and this association showed great conformity to the pathological development of LF and the intervention effects of ACF. Among these, GCDCA, a pivotal metabolite involved in the primary bile acid biosynthesis, exhibited a markedly positive correlation with core proteins such as CD44 and ITGAV in the ECM-receptor interaction pathway. High expression of CD44 and ITGAV is a key marker of HSC activation and excessive ECM deposition; by downregulating the expression of these proteins while simultaneously regulating glycochenodeoxycholic acid levels, ACF achieves dual regulation of protein-mediated cell activation and metabolism-mediated bile acid homeostasis, thereby alleviating liver injury and fibrotic deposition. Dulcitol, as a key metabolite in the fructose and mannose metabolic pathways, showed a significant negative correlation with core target proteins such as FN1 and AKT1 (Zhong et al., 2023). By inhibiting the expression of FN1 and AKT1 while simultaneously upregulating galactitol levels, ACF improves the disrupted glucose metabolism associated with LF; the normalization of glucose metabolism, in turn, further suppresses the proliferation and activation of HSCs, creating a positive feedback loop of “protein regulation—metabolic improvement—pathological relief.” These findings provide systematic molecular biological evidence elucidating the multi-target, multi-pathway mechanisms of ACF.

Despite these promising findings, several challenges remain before ACF can be translated into a clinically applicable antifibrotic agent. As a multi-component flavonoid fraction, ACF requires rigorous chemical standardization to ensure batch-to-batch consistency and reproducible efficacy. In addition, the generally low oral bioavailability of flavonoids may limit systemic exposure, highlighting the need for pharmacokinetic characterization and formulation strategies to improve solubility, stability, and absorption. Further studies should also evaluate long-term safety, dose–response relationships, and potential herb–drug interactions. Establishing links among standardized chemical profiles, pharmacokinetic exposure, and antifibrotic efficacy will be essential for the future pharmaceutical development of ACF (Figure 14).

## 5 Conclusion

This work systematically elucidated the anti-LF effect of ACF and its dual protein-metabolic regulatory mechanism. ACF significantly reduced the severity of CCl_4_-induced LF in rats, improved liver function, inhibited HSC activation, and downregulated α-SMA expression. Proteomics and inhibitor validation studies indicated that ACF elicited antifibrotic activity via regulating three core pathways—ECM-receptor interactions, FA, and the PI3K/AKT pathway—to downregulate the expression and phosphorylation levels of key proteins such as CD44, ITGAV, FN1, FAK, and AKT1. This mechanism blocked the abnormal activation of the FAK/AKT1 signaling axis, thereby inhibiting HSC proliferation and activation. Metabolomics analysis revealed that ACF significantly reversed the metabolic dysregulation induced by LF. The differentially regulated metabolites were primarily enriched in pathways such as glutathione metabolism, primary bile acid biosynthesis, and fructose and mannose metabolism, thereby alleviating oxidative stress and improving energy metabolism disorders. Multi-omics integrated analysis further constructed an association network linking differentially expressed proteins, differentially expressed metabolites, and core pathways, revealing ACF’s dual regulatory role in protein-mediated cell activation and metabolism-mediated bile acid homeostasis and glucose metabolism dysregulation, thereby forming a positive feedback loop of “protein regulation–metabolic improvement–pathological relief”. In summary, by inhibiting FAK/AKT1 signaling-dependent HSC activation and synergistically regulating key metabolic pathways, ACF exerted its anti-fibrotic effects at both the protein and metabolic levels. It represented a potential multi-target, multi-pathway therapeutic candidate for the treatment of LF.

## Data Availability

The original contributions presented in the study are included in the article/Supplementary Material, further inquiries can be directed to the corresponding authors.
