# Supplementary material for: Integrated proteomic and metabolomic analysis reveals the mechanism of astragalus complanatus flavonoids against liver fibrosis in rats
Source: Front Pharmacol. 2026 Sep 28;17:1949321. doi: 10.3389/fphar.2026.1949321 (PMC13654702; doi:10.3389/fphar.2026.1949321)
Supplement: Supplementary file 1 [file Table1.doc]

**TABLE S1 Acetonitrile-0.1% Formic Acid Water Gradient Elution Table**

| Time/min | Acetonitrile /% | 0.1% Formic Acid /% |
| --- | --- | --- |
| 0 | 5 | 95 |
| 4 | 9 | 91 |
| 8 | 16 | 84 |
| 15 | 17 | 83 |
| 24 | 21 | 79 |
| 30 | 40 | 60 |
| 34 | 95 | 5 |
| 37 | 95 | 5 |
| 38 | 9 | 91 |
| 40 | 5 | 95 |
